# Tissue plasminogen activator-treated patients with acute ischemic stroke in the pioneer public service of Rio de Janeiro: a comparative profile with the NINDS study

**DOI:** 10.1186/cc10196

**Published:** 2011-06-22

**Authors:** H Missaka, JE Almeida, PC Figueiredo, CL Nogueira, VR Julião, JLL Alencar, RS Lannes, SL Fernandes, G Caetani, J Abrantes, TD Bevilacqua, V Antonucci, PHCF Pinto, S Divan-Filho

**Affiliations:** 1 CTI2 - Hospital Municipal Souza Aguiar, Rio de Janeiro - RJ, Brazil; 2 Gama Filho University School of Medicine, Rio de Janeiro - RJ, Brazil; 3 Souza Marques School of Medicine, Rio de Janeiro - RJ, Brazil; 4 Rio de Janeiro State University School of Medicine, Rio de Janeiro - RJ, Brazil

## Introduction

Strokes are the leading cause of death in Brazil, with an incidence of 108/100,000 inhabitants [1], 31% lethality, and beyond they are causes of disability and high social costs. The National Institute of Neurological Disorders and Stroke study (NINDS) [2] and the 3rd European Cooperative Acute Stroke Study [3] demonstrated that intravenous tissue plasminogen activator (t-PA) improved clinical outcome at 3 months. This study recognized the patient's profile attending a pioneer public Stroke Team - trained at Albert Einstein and Mãe de Deus Hospitals - comparing and analyzing its results with NINDS.

## Objective

To evaluate the profile - age, door-to-needle time (Dt), NIHSS and mortality - in patients with acute ischemic stroke (AIS) treated with t-PA in Hospital Municipal Souza Aguiar (HMSA). To compare the results with NINDS' reference data.

## Methods

An observational series and analysis of cases treated with t-PA on HMSA. A review of recent literature.

## Results

From May 2006 through November 2010, 71 patients received t-PA therapy and underwent this study (Table 1). Comparing with NINDS (on average) we obtained: age: HMSA = 61.8 years (18 to 88), NINDS = 67 years. Dt HMSA = 2.65 hours (1 to 5.5). Patients obtained treatment within 1.5 hours (Dt <1.5 hours): HMSA = 11 (16%); NINDS = 71 (49%).

**Table 1:** 

	HMSA (*n *= 71)	NINDS (*n *= 177)
Age (years)	61.8 (18 to 88)	67
Dt <1.5 hours (patients)	11 (15.49%)	71 (49%)
NIHSS at admission	13,4 (5 to 24)	14 (1 to 37)
Mortality	9 (13%)	24 (17%)

## Conclusions

The study reported an early presentation of AIS, which may be associated with difficult access to primary care in this city. The entry NIHSS was similar in both studies. In the NINDS, 50% of the patients received t-PA within 1.5 hours, and only 16% in the HMSA at this time. Pre-hospital quick reference and rapid diagnosis in the emergency room could diminish the Dt. Symptomatic hemorrhage (13% HMSA) was similar if we take into account only deaths from the use of t-PA therapy. Finally, we demonstrated benefits with t-PA treatment in AIS in Rio de Janeiro and recognized limitations that, when overcome, will allow improving the treatment of such severe disorder.
